# Some Mechanical Constraints to the Biomimicry with Peripheral Nerves

**DOI:** 10.3390/biomimetics8070544

**Published:** 2023-11-13

**Authors:** Pier Nicola Sergi

**Affiliations:** Translational Neural Engineering Area, The Biorobotics Institute and Department of Excellence in Robotics and AI, Sant’Anna School of Advanced Studies, 56127 Pisa, Italy; piernicola.sergi@santannapisa.it or pn.sergi@cauchyinstitute.it

**Keywords:** biomimicry, nerve biomechanics, peripheral nerves, numerical optimization, strain energy function

## Abstract

Novel high technology devices built to restore impaired peripheral nerves should be biomimetic in both their structure and in the biomolecular environment created around regenerating axons. Nevertheless, the structural biomimicry with peripheral nerves should follow some basic constraints due to their complex mechanical behaviour. However, it is not currently clear how these constraints could be defined. As a consequence, in this work, an explicit, deterministic, and physical-based framework was proposed to describe some mechanical constraints needed to mimic the peripheral nerve behaviour in extension. More specifically, a novel framework was proposed to investigate whether the similarity of the stress/strain curve was enough to replicate the natural nerve behaviour. An original series of computational optimizing procedures was then introduced to further investigate the role of the tangent modulus and of the rate of change of the tangent modulus with strain in better defining the structural biomimicry with peripheral nerves.

## 1. Introduction

Biomimetic materials are able to behave similar to materials originating from living organisms and can effectively replicate selected characteristics of natural materials. They could replace biological materials, restoring natural functions when the biological counterparts are unpaired, absent, or unable to correctly perform [1]. Biomimetic materials were often used to build scaffolds promoting the regeneration of the natural tissue: biomimetic 3D-printed scaffolds for spinal cord injury repair were built [2], as well as electrically conductive scaffolds mimicking the hierarchical structure of cardiac myofibers [3]. Again, in the literature, biomimetic scaffolds for tendon tissue regeneration were described [4,5,6,7]. More generally, some research groups focused on biomimetic approaches for bone tissue engineering [8,9,10], while others obtained a supramolecular biomimetic skin [11]. Some scientists succeeded in building a biomimetic nerve platform using cell line as neuronal population [12,13], while others provided some general design principles for a biomimetic artificial nerve [14]. More specifically, as “desired biomimetic devices and scaffolds for neurotrophic biomolecules to be implemented in a future PNI-repair scaffold”, they described a three-dimensional scaffold architecture designed to improve the regeneration of sensory and motor fibers. The internal channels were described as able to guide a very small group of axons through scaffolds, which were biofunctionalized to bind the receptors expressed at the surface of the growth cone. Interestingly, all the previous structures were enveloped by a material, which formed the outer sheet of the biomimetic artificial nerve, and which was identified as “semipermeable”, “semirigid”, or “flexible”. Nevertheless, the mechanical characteristics of this “material” were not described and are not currently clear, since it should mimic the mechanics of the natural outer sheet of connective tissue enveloping peripheral nerves. In other words, the knowledge of the peripheral nerve mechanical response to axial strain was needed to allow this material to be properly designed. Indeed, peripheral nerves are physical objects [15], which react to elongation by increasing their stiffness to keep the axons integrity and to protect endoneural structures from longitudinal overstretch [16]. The study of the mechanical behaviour of peripheral nerves was performed at different scale levels. Thus, the relationship between macroscopic tensile loads and micro-scale deformations [17] was investigated. Again, the nerve was studied as a complex structure showing the characteristics of an isotropic material, providing a deterministic elastic [18,19] or hyperelastic response [20,21,22,23,24] as a function of the strain magnitude. Differently, a stochastic iterative fibril-scale mechanical model was implemented to reproduce the straightening of wavy fibrils and to account for the effects of interfibrillar crosslinks on the overall properties of the tissue [25,26]. Recently, an explicit and deterministic framework was proposed to model the behaviour of peripheral nerves under longitudinal stretch [27]. This framework was applied here to investigate the main mechanical constraints to the behaviour of a material, which could mimic the axial response of peripheral nerves. The governing equations were proposed in polynomial form together with an original procedure to decrease their overall complexity in physiologically relevant cases. Finally, the minimum number of constraints to achieve an acceptable level of biomimicry with peripheral nerves was presented.

## 2. Methods

The mechanical response of the peripheral nervous tissue was assumed to be ruled by an SEF (Strain Energy Function, for unit of volume) affected by the strain measure as well as by the initial direction of the collagen fibrils. In particular, the SEF was written as W=W(C,M⊗M), where F was the deformation gradient and $C=F^{T}F$ was the right Cauchy–Green strain tensor, while M was a unit vector field accounting for the direction of the collagen fibrils in the reference configuration. The mean Cauchy–Green stress tensor was, then, written as:

(1) $$\sigma =J^{-1}F\frac{\partial W}{\partial F}$$

and J=det(F). In general, the strain energy function could be written as a function of invariants as $W=W(I_{1},I_{2},I_{3},I_{4},I_{5})$, where, $I_{1}=tr\left(C\right)$, $I_{2}=\frac{1}{2}\left[I_{1}^{2}-tr\left(C^{2}\right)\right]$, $I_{3}=det\left(C\right)$, $I_{4}=M\cdot \left(\mathrm{CM}\right)$, $I_{5}=M\cdot \left(C^{2}M\right)$. As usual, the peripheral nervous tissue was considered incompressible, and thus, $I_{3}=det\left(C\right)=1$ [16,22,23,24,28,29]. In addition, since its main structural components are the ground matrix [30,31] and the collagen fibrils [32], the strain energy function was rewritten as $W=W(I_{1},I_{4})$, where the $I_{1}$ invariant accounted for the action of the matrix, and the $I_{4}$ invariant modeled the reinforcing action of the collagen fibrils. Therefore, the mean Cauchy–Green stress tensor was:

(2) $$\sigma =-\tilde{p}I+2W_{1}B+2W_{4}m⊗m$$

where $\tilde{p}$ was the pressure, $B={\mathrm{FF}}^{T}$ was the left Cauchy–Green tensor, $W_{i}=\frac{\partial W}{\partial I_{i}}$, i=1,4, and m=FM. In particular, the energetic contributions, deriving from the ground matrix and from the collagen fibrils were chosen, as in the recent literature [27].

The nerve underwent to axial strain (z-direction), and thus, its lateral surface was stress-free. Then, the determination of the mean axial stress as a function of the axial strain ($\epsilon_{zz}$) was performed through Equation (2) solved with the boundary conditions $\sigma_{xx}=\sigma_{yy}=0$ [28,29]. A polynomial form of the mean Cauchy stress was, then, written as:

(3) $$\sigma_{zz}\left(K_{1},A,D,p,q,\epsilon_{zz}\right)=2K_{1}\left[{\left(\epsilon_{zz}+1\right)}^{2}-\sum_{n=0}^{p}{\left(-1\right)}^{n}\epsilon_{zz}^{n}\right]+4AD\epsilon_{zz}\left(2+\epsilon_{zz}\right)\left\{\sum_{n=0}^{q}\frac{{\left[D\epsilon_{zz}^{2}{\left(2+\epsilon_{zz}\right)}^{2}\right]}^{n}}{n!}-1\right\}$$

the tangent modulus was written as:

(4) $$\begin{array}{c} E_{t}\left(K_{1},A,D,p,q,\epsilon_{zz}\right)=\left(4K_{1}-8AD\right)\left(\epsilon_{zz}+1\right)-2K_{1}\sum_{n=0}^{p}n{\left(-1\right)}^{n}\epsilon_{zz}^{\left(n-1\right)}+8AD\left(\epsilon_{zz}+1\right)\sum_{n=0}^{q}\frac{D^{n}\epsilon_{zz}^{2n}{\left(\epsilon_{zz}+2\right)}^{2n}}{n!}+\\ +4AD\epsilon_{zz}\left(\epsilon_{zz}+2\right)\sum_{n=0}^{q}\frac{\epsilon_{zz}^{2n}{\left(\epsilon_{zz}+2\right)}^{2n}\left(4D^{n}n\epsilon_{zz}+4D^{n}n\right)}{n!\epsilon_{zz}^{2}+2n!\epsilon_{zz}} \end{array}$$

while the rate of change of the tangent modulus with strain was:

(5) $$\begin{array}{c} E_{t}^{\prime }\left(K_{1},A,D,p,q,\epsilon_{zz}\right)=4K_{1}-8AD-2K_{1}\sum_{n=0}^{p}\left[\left(n^{2}-n\right){\left(-1\right)}^{n}\epsilon_{zz}^{n-2}\right]+8AD\sum_{n=0}^{q}\frac{\left(D^{n}\epsilon_{zz}^{2n}{\left(\epsilon_{zz}+2\right)}^{2n}\right)}{n!}+\\ +4AD\epsilon_{zz}\left(\epsilon_{zz}+2\right)\sum_{n=0}^{q}\frac{\epsilon_{zz}^{2n}{\left(\epsilon_{zz}+2\right)}^{2n}\left(\left(16D^{n}n^{2}-4D^{n}n\right)\epsilon_{zz}^{2}+\left(32D^{n}n^{2}-8D^{n}n\right)\epsilon_{zz}+16D^{n}n^{2}-8D^{n}n\right)}{\left(n!\epsilon_{zz}^{4}+4n!\epsilon_{zz}^{3}+4n!\epsilon_{zz}^{2}\right)}+\\ +16AD\left(\epsilon_{zz}+1\right)\sum_{n=0}^{q}\frac{\epsilon_{zz}^{2n}{\left(\epsilon_{zz}+2\right)}^{2n}\left(4D^{n}n\epsilon_{zz}+4D^{n}n\right)}{\left(n!\epsilon_{zz}^{2}+2n!\epsilon_{zz}\right)} \end{array}$$

In Equations (3)–(5) the indexes p,q∈N should be determined in order to minimize the functional:

(6) $$\Phi \left(K_{1},A,D,p,q\right)=\int_{0}^{\epsilon_{zz_{max}}}\left|\sigma_{zz}\left(K_{1},A,D,p,q,\epsilon_{zz}\right)-\sigma_{zz}\left(K_{1},A,D,\infty ,\infty ,\epsilon_{zz}\right)\right|d\epsilon_{zz}$$

where $\sigma_{zz}\left(K_{1},A,D,\infty ,\infty ,\epsilon_{zz}\right)=\lim_{p\to \infty ,q\to \infty }\sigma_{zz}\left(K_{1},A,D,p,q,\epsilon_{zz}\right)$. The sensitivity of the mean Cauchy stress in Equation (3) to each parameter was evaluated through the following indexes:

(7) $$\begin{array}{r} S_{K_{1}}\left(1+\Delta K_{1},p,q\right)=\frac{\int_{0}^{\epsilon_{zz_{max}}}\left|\sigma_{zz}\left(1+\Delta K_{1},1,1,p,q,\epsilon_{zz}\right)-\sigma_{zz}\left(1,1,1,p,q,\epsilon_{zz}\right)\right|d\epsilon_{zz}}{\Delta K_{1}} \end{array}$$

(8) $$\begin{array}{r} S_{A}\left(1+\Delta A,p,q\right)=\frac{\int_{0}^{\epsilon_{zz_{max}}}\left|\sigma_{zz}\left(1,1+\Delta A,1,p,q,\epsilon_{zz}\right)-\sigma_{zz}\left(1,1,1,p,q,\epsilon_{zz}\right)\right|d\epsilon_{zz}}{\Delta A} \end{array}$$

(9) $$\begin{array}{r} S_{D}\left(1+\Delta D,p,q\right)=\frac{\int_{0}^{\epsilon_{zz_{max}}}\left|\sigma_{zz}\left(1,1,1+\Delta D,p,q,\epsilon_{zz}\right)-\sigma_{zz}\left(1,1,1,p,q,\epsilon_{zz}\right)\right|d\epsilon_{zz}}{\Delta D} \end{array}$$

where $\Delta K_{1}$ = ΔA = ΔD=99. In addition, the best combination of the parameters p,q was chosen through the maximization of the metric:

(10) $$\Omega \left(K_{1},A,D,p,q\right)=\frac{|log\left[\Phi \left(K_{1},A,D,p,q\right)\right]{|}^{1.7}}{\left(p+1)(q+1\right)}$$

## 3. Results

### 3.1. Error Evaluation for Different Polynomial Stress Functions

**Figure 1:**
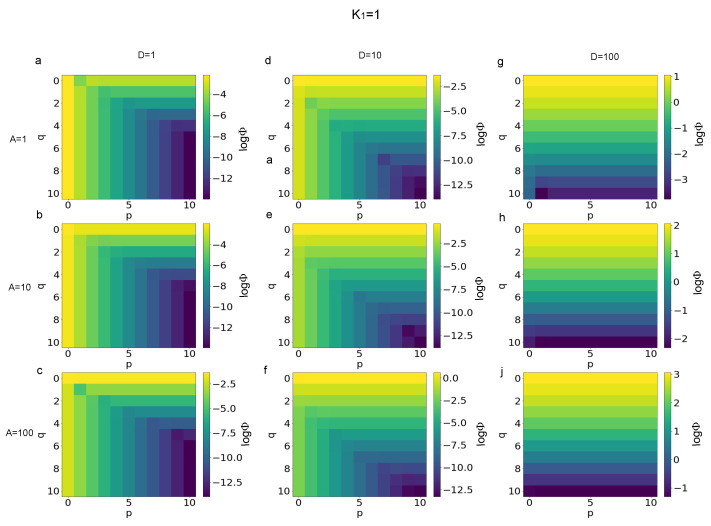
Magnitude of errors (*log*Φ) for $K_{1}=1$ and different ranges of *A* and *D* parameters. (**a**–**c**) Magnitude of errors for *D* = 1 and *A* = 1, 10, 100, respectively. (**d**–**f**) Magnitude of errors for *D* = 10 and *A* = 1, 10, 100. (**g**,**h**,**j**) Magnitude of errors for *D* = 100 and *A* = 1, 10, 100, respectively.

**Figure 2:**
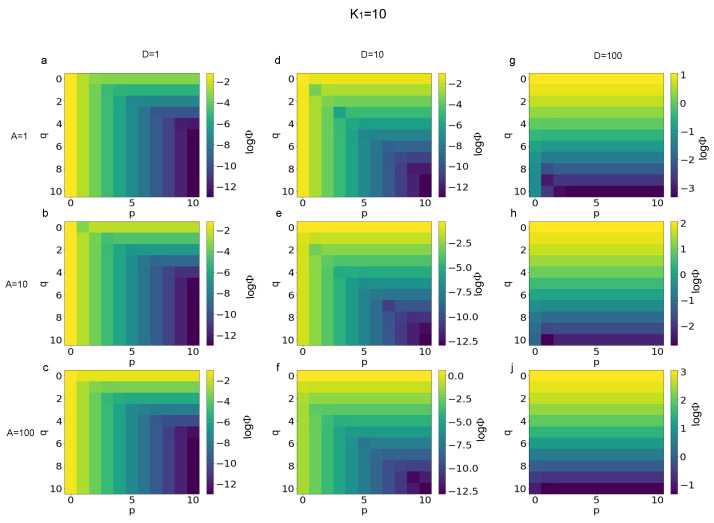
Magnitude of errors (*log*Φ) for $K_{1}=10$ and different ranges of *A* and *D* parameters. (**a**–**c**) Magnitude of errors for *D* = 1 and *A* = 1, 10, 100, respectively. (**d**–**f**) Magnitude of errors for *D* = 10 and *A* = 1, 10, 100. (**g**,**h**,**j**) Magnitude of errors for *D* = 100 and *A* = 1, 10, 100, respectively.

**Figure 3:**
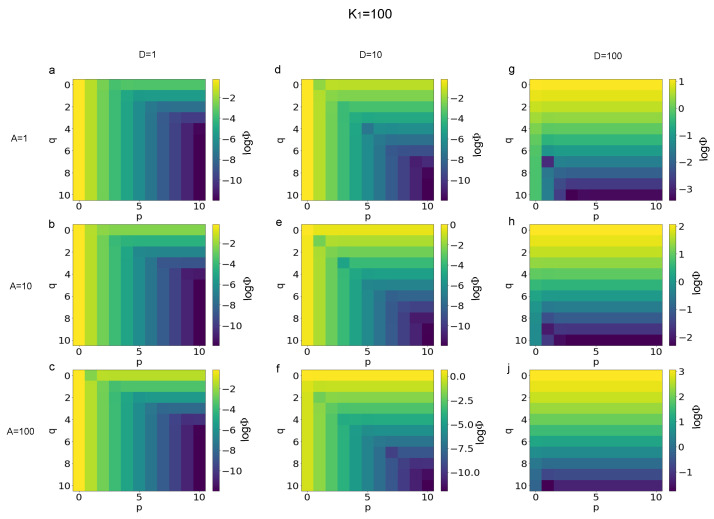
Magnitude of errors (*log*Φ) for $K_{1}=100$ and different ranges of *A* and *D* parameters. (**a**–**c**) Magnitude of errors for *D* = 1 and *A* = 1, 10, 100, respectively. (**d**–**f**) Magnitude of errors for *D* = 10 and *A* = 1, 10, 100. (**g**,**h**,**j**) Magnitude of errors for *D* = 100 and *A* = 1, 10, 100, respectively.

The logarithm (base 10) of the functional defined in Equation (6) was used to quantify the error of Equation (3) with respect to the limit values of p→∞ and q→∞ for each combination of finite values for *p* and *q*. More specifically, in Figure 1 the magnitude of errors is shown for $K_{1}=1$ and different values of the *A* and *D*. In particular, the value of logΦ is shown for D=1 and A=1,10,100 (Figure 1a–c), for D=10 and A=1,10,100 (Figure 1d–f), and for D=100 and A=1,10,100 (Figure 1g–j). Similarly, in Figure 2, the error magnitude is shown for $K_{1}=10$. More specifically, the magnitude of errors is shown for D=1 and A=1,10,100 (Figure 2a–c), for D=10 and A=1,10,100 (Figure 2d–f), and for D=100 and A=1,10,100 (Figure 2g–j). In the same way, in Figure 3 the magnitude of the errors are shown for $K_{1}=100$. The values are shown for D=1 and A=1,10,100 (Figure 3a–c), for D=10 and A=1,10,100 (Figure 3d–f), and, finally, for D=100 and A=1,10,100 (Figure 3g–j).

### 3.2. Sensitivity of Different Polynomial Stress Functions to Parameters $K_{1},A,D$

**Figure 4:**
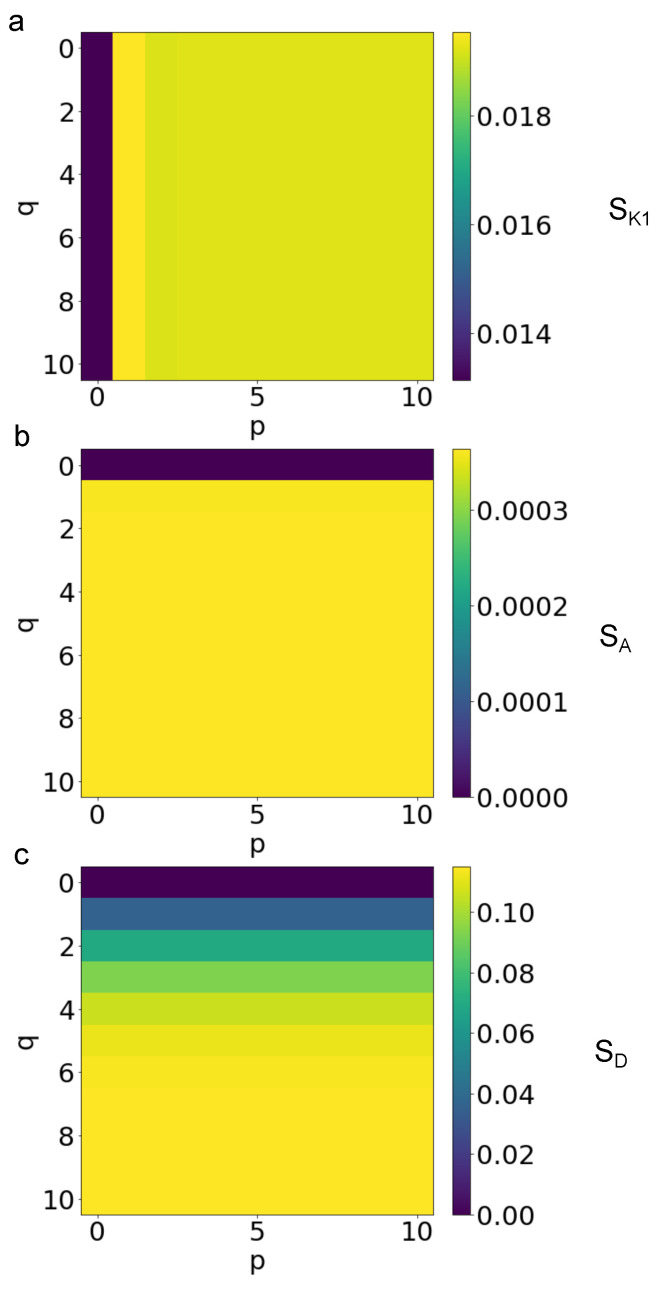
Sensitivity of Equation (3) to parameters $K_{1},A,D$. (**a**) Sensitiveness of Equation (3) to the increment of $K_{1}$ from 1 to 100 (A=1,D=1). (**b**) Sensitivity of Equation (3) to the increment of *A* from 1 to 100 ($K_{1}=1$ and D=1). (**c**) Sensitivity of Equation (3) to the increment of *D* from 1 to 100 ($K_{1}=1$ and A=1).

The sensitivity of Equation (3) to parameters $K_{1},A,D$ was studied through Equations (7)–(9). More specifically, in Figure 4a, the influence of the change of $K_{1}$ from 1 to 100 was studied for all combinations of 0≤p≤10 and 0≤q≤10, when both the other parameters were kept constant (i.e., A=1,D=1). The value of sensitivity $S_{K_{1}}$ (for sake of simplicity all the dependencies from parameters were dropped out) ranged from $1.314\times 10^{-2}$ to $1.954\times 10^{-2}$. In the first column, there were the minimum values, while in the second column there were the maximum ones. The value of sensitivity was almost constant (i.e., $S_{K_{1}}$ oscillated around $1.192\times 10^{-2}$) over all the other combinations. Similarly, in Figure 4b, the influence of the change of the parameter *A* from 1 to 100 was studied, keeping constant both $K_{1}=1$ and D=1. In this case, the sensitivity $S_{A}$ ranged between 0 and $3.635\times 10^{-4}$. The fist row was the minimum one, the value of sensitivity was $S_{A}=3.602\times 10^{-4}$ in the second row, while its value was almost constant (i.e., $S_{A}=3.635\times 10^{-4}$) over all the other combinations. Finally, in Figure 4c, the influence of the change of the parameter *D* from 1 to 100 was studied, while the other parameters were constant $K_{1}=1$ and A=1. The sensitivity $S_{D}$ ranged between 0 and $11.503\times 10^{-2}$. In this case, the sensitivity increased with the value of *q*. The fist row was the minimum one, while the value of $S_{D}$ progressively increased from $3.638\times 10^{-2}$ to $11.503\times 10^{-2}$ starting from the 2nd row to the 10th one.

### 3.3. Minimization of the p and q Values

**Figure 5:**
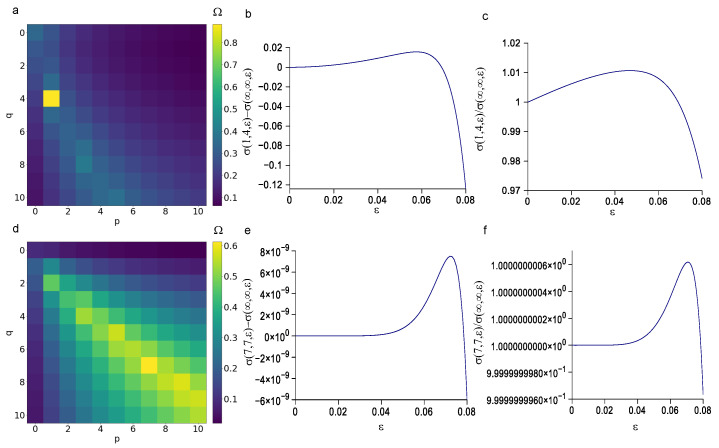
Suitability assessment of combinations with finite values of *p* and *q*. (**a**) The metric Ω identified as best candidate the stress function with p=1 and q=4 (in yellow) for the peroneal nerve. (**b**) Evolution of the difference between stress functions with p=1, q=4, and p=∞, q=∞. (**c**) Evolution of the ratio between stress functions with p=1, q=4, and p=∞, q=∞. (**d**) The metric Ω identified as best candidate the stress function with p=7 and q=7 (in yellow) for the vagus nerve. (**e**) Evolution of the difference between stress functions with p=7, q=7, and p=∞, q=∞. (**f**) Evolution of the ratio between stress functions with p=7, q=7, and p=∞, q=∞. In all relevant plots $\epsilon_{zz}=\epsilon$ for sake of simplicity.

Once studied, the sensitivity of Equation (3) to general values of parameters $K_{1},A,D$, the attention was narrowed to their physiological values. In particular, literature values were used both for peroneal and vagus nerves [27]. The metric Ω in Equation (10) was used to identify the best combination of parameters (with finite and integer values ranging between 0 and 10) for Equation (3) with physiological parameters for peroneal nerve ($K_{1}=3.018$ kPa, A=0.014 kPa, and D=66.893). The maximization of this metric resulted in Ω=0.883 for p=1 and q=4. Therefore, to further compare the evolution of $\sigma_{zz}\left(1,4,\epsilon \right)$ to the $\sigma_{zz}\left(\infty ,\infty ,\epsilon \right)$ (the dependence of parameters was dropped out for sake of simplicity), both their difference and their ratio were studied. More specifically, their difference started from 0 and increased up to $1.57\times 10^{-2}$ kPa for ϵ=0.058, while it decreased up to $-1.24\times 10^{-1}$ for ϵ=0.08 (Figure 5b). Similarly, their ratio started form 1 for ϵ=0 and increased up to 1.0000033 for ϵ=0.069, while it decreased up to 0.974 for ϵ=0.08 (Figure 5c). In a similar way, the maximization of the metric in Equation (10) resulted in Ω=0.612 for p=7 and q=7 when the mean physiological value for vagus nerve were used ($K_{1}=9.731$ kPa, A=5.277 kPa, D=10.171). The difference between $\sigma_{zz}\left(7,7,\epsilon \right)$ and $\sigma_{zz}\left(\infty ,\infty ,\epsilon \right)$ (the dependence of parameters was dropped out for sake of simplicity) started from 0 for ϵ=0 and increased up to $7.183\times 10^{-9}$ for ϵ=0.074, while it decreased up to $-5.955\times 10^{-9}$ for ϵ=0.08 (Figure 5e). Their ratio started from 1 for ϵ=0 and oscillated from 1.0000000006 for ϵ=0.071 and 0.9999999996 for ϵ=0.08 (Figure 5f).

### 3.4. Evolution of Stress, Tangent Modulus, and Rate of Change of the Tangent Modulus with Strain

**Figure 6:**
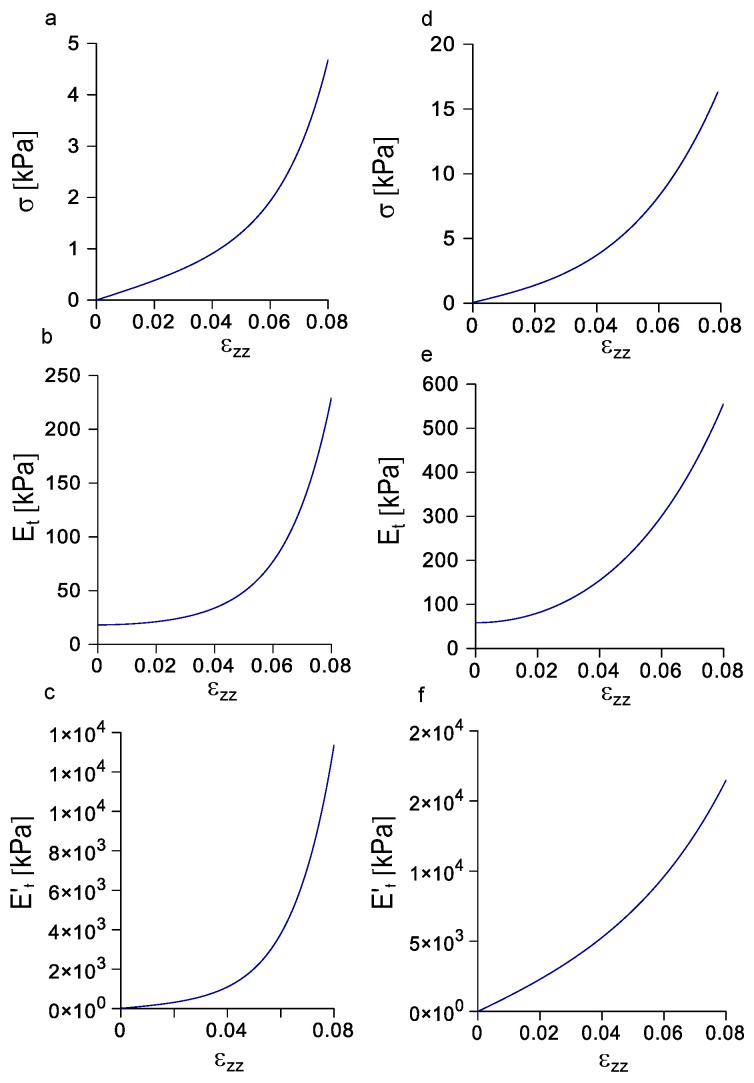
Evolution of stress, tangent modulus, and rate of change of tangent modulus with strain. (**a**) Evolution of the mean Cauchy stress with strain for the porcine peroneal nerve. (**b**) Evolution of the tangent modulus with strain for the porcine peroneal nerve. (**c**) Evolution of the rate of change of the tangent modulus with strain for the porcine peroneal nerve. (**d**) Evolution of the mean Cauchy stress with strain for the canine vagus nerve. (**e**) Evolution of the tangent modulus with strain for the canine vagus nerve. (**f**) Evolution of the rate of change of the tangent modulus with strain for the canine vagus nerve.

The analysis performed through Equation (10) was able to identify the best combination of finite values of parameters *p* and *q* with physiological parameters. In particular, the evolution of Equation (3) with $K_{1}=3.018$ kPa, A=0.014 kPa, D=66.893, p=1, and q=4 is shown in Figure 6a for the porcine peroneal nerve. The mean Cauchy stress increased monotonically and non linearly starting from 0 kPa for ϵ=0 up to 4.676 kPa for ϵ=0.08. Similarly, the evolution of tangent modulus was non linearly increasing from 18.018 kPa for ϵ=0 to 229.125 kPa for ϵ=0.08 (Figure 6b), while the rate of change of the tangent modulus with strain started from 12.072 for ϵ=0 and non linearly increased up to 13,357.327 for ϵ=0.08 (Figure 6c). Furthermore, the evolution of Equation (3) with $K_{1}=9.731$ kPa, A=5.277 kPa, D=10.171, p=7, and q=7 is shown in Figure 6d for the canine vagus nerve. The Cauchy stress increased monotonically in a non linear way starting from 0 kPa for ϵ=0 up to 16.300 kPa for ϵ=0.08, while the tangent modulus non linearly increased from 58.386 kPa for ϵ=0 to 555.722 kPa for ϵ=0.08 (Figure 6e). Finally, the rate of change of the tangent modulus with strain started from 0 for ϵ=0 and non linearly increased up to 16,479.238 for ϵ=0.08 (Figure 6f).

### 3.5. Analysis of the Correlation among Longitudinal Stress, Tangent Modulus, and Rate of Change of the Tangent Modulus with Strain

**Figure 7:**
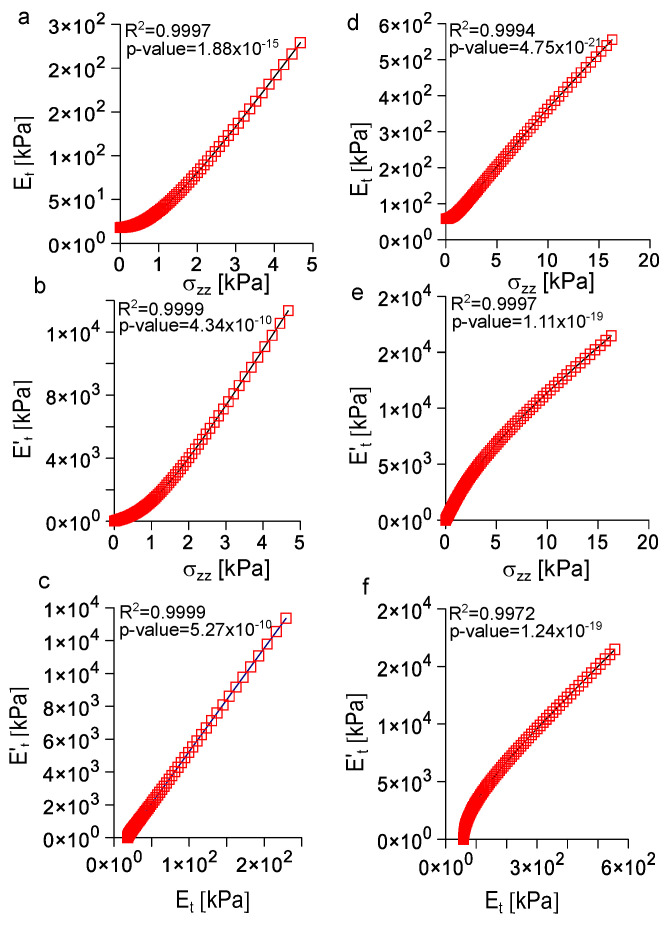
Correlation between relevant quantities for peroneal and vagus nerves. (**a**) Correlation between the mean Cauchy stress and the tangent modulus for the peroneal nerve ($R^{2}=0.9997$, *p*-value = $1.88\times 10^{-15}$, α=0.05, two-tailed *t* test). (**b**) Correlation between the mean Cauchy stress and the change of the tangent modulus with strain for the peroneal nerve ($R^{2}=0.9999$, *p*-value = $4.34\times 10^{-10}$, α=0.05, two-tailed *t* test). (**c**) Correlation between the tangent modulus andthe change of the tangent modulus with strain for the peroneal nerve ($R^{2}=0.9999$, *p*-value = $5.27\times 10^{-10}$, α=0.05, two-tailed *t* test). (**d**) Correlation between the mean Cauchy stress and the tangent modulus for the vagus nerve ($R^{2}=0.9994$, *p*-value = $4.75\times 10^{-21}$, α=0.05, two-tailed *t* test). (**e**) Correlation between the mean Cauchy stress and the change of the tangent modulus with strain for the vagus nerve ($R^{2}=0.9997$, *p*-value = $1.11\times 10^{-19}$, α=0.05, two-tailed *t* test). (**f**) Correlation between the tangent modulus and the change of the tangent modulus with strain for the vagus nerve ($R^{2}=0.9972$, *p*-value = $1.24\times 10^{-19}$, α=0.05, two-tailed *t* test).

In order to investigate possible relationships among the previously described quantities, a further analysis is shown in Figure 7. More specifically, in Figure 7a, the evolution of the mean Cauchy stress with strain was compared to the evolution of the tangent modulus with strain for the porcine peroneal nerve. These quantities were positively correlated ($R^{2}=0.9997$, *p*-value = $1.88\times 10^{-15}$, α=0.05, two-tailed *t* test) through the non linear expression:

(11) $$E_{t}\left(1,4,\epsilon_{zz}\right)=-1.574\sigma_{zz}^{3}\left(1,4,\epsilon_{zz}\right)+15.548\sigma_{zz}^{2}\left(1,4,\epsilon_{zz}\right)+6.575\sigma_{zz}\left(1,4,\epsilon_{zz}\right)+16.839$$

Similarly (Figure 7b), the rate of change of the tangent modulus with strain was positively correlated ($R^{2}=0.9999$, *p*-value = $4.34\times 10^{-10}$, α=0.05, two-tailed *t* test) to the mean Cauchy stress through the non linear formula:

(12) $$E_{t}^{\prime }\left(1,4,\epsilon_{zz}\right)=-93.852\sigma_{zz}^{3}\left(1,4,\epsilon_{zz}\right)+943.702\sigma_{zz}^{2}\left(1,4,\epsilon_{zz}\right)+468.751\sigma_{zz}\left(1,4,\epsilon_{zz}\right)+4.126$$

Again (Figure 7c), the evolution of the rate of change of the tangent modulus with strain was positively correlated ($R^{2}=0.9999$, *p*-value = $5.27\times 10^{-10}$, α=0.05, two-tailed *t* test) to the tangent modulus through the linear expression:

(13) $$E_{t}^{\prime }\left(1,4,\epsilon_{zz}\right)=62.838E_{t}\left(1,4,\epsilon_{zz}\right)-1036.407$$

In Equations (11)–(13), the explicit dependence of $K_{1}=3.018$ kPa, A=0.014 kPa, and D=66.893 was neglected for sake of simplicity. In a similar way (Figure 7d), for the canine vagus nerve, the evolution of the tangent modulus was positively correlated with the longitudinal strain ($R^{2}=0.9994$, *p*-value = $4.75\times 10^{-21}$, α=0.05, two-tailed *t* test) through the non linear expression:

(14) $$E_{t}\left(7,7,\epsilon_{zz}\right)=-0.036\sigma_{zz}^{3}\left(7,7,\epsilon_{zz}\right)+0.800\sigma_{zz}^{2}\left(7,7,\epsilon_{zz}\right)+27.432\sigma_{zz}\left(7,7,\epsilon_{zz}\right)+47.594$$

The rate of change of the tangent modulus (Figure 7e) was positively correlated ($R^{2}=0.9997$, *p*-value = $1.11\times 10^{-19}$, α=0.05, two-tailed *t* test) to the longitudinal stress through the non linear expression:

(15) $$E_{t}^{\prime }\left(7,7,\epsilon_{zz}\right)=2.442\sigma_{zz}^{3}\left(7,7,\epsilon_{zz}\right)-81.481\sigma_{zz}^{2}\left(7,7,\epsilon_{zz}\right)+1694.064\sigma_{zz}\left(7,7,\epsilon_{zz}\right)+130.716$$

Finally, the evolution of the rate of change of the tangent modulus with strain was positively correlated ($R^{2}=0.9972$, *p*-value = $1.24\times 10^{-19}$, α=0.05, two-tailed *t* test) with the evolution of the tangent modulus (Figure 7f) with the non linear relationship:

(16) $$E_{t}^{\prime }\left(7,7,\epsilon_{zz}\right)=0.000116E_{t}^{3}\left(7,7,\epsilon_{zz}\right)-0.124E_{t}^{2}\left(7,7,\epsilon_{zz}\right)+68.524E_{t}\left(7,7,\epsilon_{zz}\right)-2788.716$$

In Equations (14)–(16), the explicit dependence of $K_{1}=9.731$ kPa, A=5.277 kPa, and D=10.171 was neglected for the sake of simplicity.

## 4. Discussion

In this work, some basic mechanical constraints were investigated to design a material biomimetic to the connective tissue of peripheral nerves. This material was anticipated as required to build a biomimetic artificial nerve [14]. However, no quantitative information was up to now provided about its mechanical characteristics. As a consequence, since the peripheral nerves are mainly exposed to axial elongations, here, the characteristic for a material to behave like peripheral nerves in extension are quantitatively explored. An original polynomial framework was also provided, in order to simplify as much as possible the writing of the governing equations. To this aim, some different numerical analyses of the behaviour of Equations (3)–(5) were performed.

A first analysis of the magnitude of errors, expressed through the logarithm of the functional $\Phi (K_{1},A,D,p,q)$ in Equation (6), was able to show that different patterns arose for different values of parameters. In other words, the distribution of errors for all the combinations of integers values of p,q∈N and 0≤p≤10, 0≤q≤10 changed as a function of $K_{1},A,D$ parameters. More specifically, for $K_{1}=1$, D=1, and q≥5 (Figure 1), the magnitude of errors depended only of *p* and decreased from about −4 to −13 when *p* increased from 0 to 10. For $K_{1}=100$ and D=100, instead, the magnitude of errors practically only depended of *q*, ranging from −1 to −4, when *q* ranged between 0 and 10, while A=1. It is worthy to notice that the error value ranged between 2 and −2 for A=10 and between 3 and −1 for A=100. For D=10 the pattern of errors was hybrid between the two previous cases. Similarly, when $K_{1}=10$ (Figure 2) for D=1 the values of the logarithm of the functional in Equation (6) were independent of *q* and ranged between about −2 and −12 when *p* increased from 0 to 10. For D=100, the values of error were practically not dependent of *p* and ranged between 1 and −3 for A=1, between 2 and −2 for A=10, while between 3 and −1 for A=100, when *p* ranged from 0 to 10. Furthermore, in this case, for D=10 the error pattern was hybrid between the two previous cases. Finally, for $K_{1}=100$ the distribution of errors was similar (see Figure 3), while the magnitude of errors (absolute value) was slightly decreased for D=1 and D=10.

This kind of analysis was useful to learn about the distribution of errors among different combinations. However, because of the previously described dependency of parameters $K_{1},A,D$ and the possible lack of dependency of *p* or *q*, several different combinations resulted in the same amount of error. As a consequence, a further sensitivity analysis was performed (see Figure 4) in order to clarify the influence of each parameter on the amount of error. In particular, the influence of the parameter $K_{1}$ was studied through the sensitivity index in Equation (7) and underlined a lack of dependency of *q* for p≥2, where the sensitivity was the same for all combinations of *p* and *q*. In a similar way, the sensitivity index defined in Equation (8) showed a lack of dependency of *q* for p≥1, where the sensitivity pattern was flat. Moreover, the sensitivity index in Equation (9) clearly showed the total lack of dependence of *p* together with the increase of *q*. In addition, in general, the order of magnitude of $S_{D}$ was 5.56 times $S_{K1}$, while it was 333.334 times $S_{A}$. Thus, the *D* parameter had the maximum influence on the evolution of Equation (3), followed by $K_{1}$ and then by the parameter *A*. However, also in this case, several combinations had the same amount of sensitivity with respect to these parameters, which means this analysis was not enough to choose the best combination of *p* and *q* for Equation (3).

As a consequence, the metric in Equation (10) was used to account for at the same time both the value of the functional in Equation (6) and the values of *p* and *q*. Indeed, from a side, this metric was directly proportional to the absolute values of the logarithm of errors, which means it was able to found the combinations resulting in small errors. From the other side, this metric was inversely proportional to the values of *p* and *q*, then it was able to identify good candidates with small values of *p* and *q*. The use of this metric resulted also dependent of the values of parameters $K_{1},A,D$. Therefore, to perform an accurate selection of the best possible combinations related to a given biological tissue, previously identified values [27] were used, both for the porcine peroneal nerve and for the canine vagus nerve. This procedure resulted in the clear selection of the values p=1 and q=4 for the porcine peroneal nerve. The maximum difference between the evolution of the mean Cauchy stress in Equation (3) with p=1 and q=4 with respect to the same equation with p→∞ and q→∞ was within the 2.66%, as well as the ratio, which was 0.974. Therefore, Equations (3)–(5) were greatly simplified in their writing, keeping a good level of accuracy. On the contrary, for the canine vagus nerve the use of the Ω metric in Equation (6), resulted in the selection of the values p=7 and q=7. In this last case, the errors for the difference and the ratio between the reduced polynomial expression and the complete one were both in the order of $1\times 10^{-9}$, while together a single best combination, some other suboptimal combinations were found, as shown in Figure 5d (diagonal values).

Once simplified forms for Equations (3)–(5) were provided, their evolution with strain was explicitly explored (Figure 6). Both for porcine peroneal nerve and canine vagus nerves, the increase of the mean Cauchy stress with strain was highly non linear (see Figure 6a,d), in accordance to the previous literature [20,21,22,23,24,27]. This phenomenon was related to the strain stiffening ability of peripheral nerves, which was likely developed to preserve the integrity of their internal structures [33,34,35,36,37,38]. However, to provide a more complete picture of the behaviour of peripheral nerves under axial elongations, the evolution of the tangent modulus was investigated. Furthermore, in this case, for both nerves, the tangent modulus evolved in a highly non linear way (see Figure 6b,e). In addition, the rate of change of the tangent modulus with strain was explored. This further analysis was able to underline the highly non linear instantaneous evolution of the tangent modulus with the strain (see Figure 6c,f). It could be worthy to notice that even if the non linear evolution of the stress with strain was quite well known in the literature [20,21,22], few works were provided to describe the evolution of the tangent modulus, as well as the evolution of the rate of change of the tangent modulus with strain [24].

Although, intuitively, a material should behave “like natural peripheral nerves” to provide a good level of structural biomimicry, it is not clear whether only the similarity of the stress/strain response is enough. To better investigate this point, a correlation analysis was performed for both the previously described responses of both the porcine peroneal and canine vagus nerves (see Figure 7). This analysis resulted in a highly non linear positive correlations between the tangent modulus and the mean Cauchy stress both for the porcine peroneal and the canine vagus nerves (see Figure 7a,d), as in Equations (11) and (14). Similarly, a positive non linear correlation was found between the rate of change of the tangent modulus and the mean Cauchy stress for both nerves (see Figure 7b,e), as in Equations (12) and (15). Finally, for the porcine peroneal nerve a linear correlation was found between the rate of change of the tangent modulus and the evolution of the tangent modulus (see Figure 7c), as in Equation (13), while for the canine vagus nerve the same correlation was non linear (see Figure 7f), as in Equation (16). The previous results supported the general need of a comparison with the complete dynamic state of the material, namely evolution of stress, tangent modulus, and rate of change of the tangent modulus with strain, to provide an acceptable level of biomimicry with peripheral nerves. The linear correlation found between the rate of change of tangent modulus and the evolution of tangent modulus for porcine peroneal nerve was likely due to the particular numeric values of parameters (i.e., $K_{1},A,D$), which were used in this work, in particular the low value of *A*. Indeed, different values of these parameters for the canine nerve were enough to show that a comparison with only two quantities (stress and tangent modulus with strain, due to the linear correlation between the rate of change of the tangent modulus and the evolution of the tangent modulus with strain) was not enough in general.

## 5. Conclusions

Peripheral nerves under stretch behave in a complex way, increasing their instantaneous stiffness. As a consequence, in this work, an original and complete study was provided to identify general and specific polynomial equations ruling the evolution of stress, tangent modulus and rate of change of tangent modulus with strain. Different analyses were provided to identify the simplest equations able to model the physiological behaviour of different nerve specimens (porcine peroneal and canine vagus nerves). The complete picture of the “static” and “dynamic” evolution of stress with strain was provided for these two different cases. The comparison with all these three quantities was shown to be the basic reference for an acceptable level of biomimicry with peripheral nerves. Indeed, material mimicking the mechanical behaviour of nerves could be strategic in established medical procedures [39,40], interactions with implantable devices [41,42], as well as in novel technological applications as neural interfaces [43,44], stretchable electronics [45], and direct connection between engineered biomaterials and nerves cells [46,47,48,49,50] or peripheral nerves [51,52]. In addition, a good level of biomimicry with peripheral nerves could be crucial to control the immune response of the nervous tissue [53,54] in response to the stiffness mismatch between nervous tissue and biomaterials [45,55]. Indeed, this mismatch is likely due to both the “static” actual difference between stress, but also to the “dynamic” evolution of this difference with strain. Therefore, the investigation of the evolution of the tangent modulus, and of the rate of change of the tangent modulus with strain could provide explicit and quantitative mechanical constraints for structural materials biomimetic to peripheral nerves.

## Data Availability

The data presented in this study are available on request from the corresponding author.
